# Antioxidant Action and *In Vivo* Anti-Inflammatory and Antinociceptive Activities of *Myrciaria floribunda* Fruit Peels: Possible Involvement of Opioidergic System

**DOI:** 10.1155/2020/1258707

**Published:** 2020-04-27

**Authors:** Izabelly Bianca da Silva Santos, Bruno Santos dos Santos, João Ricardhis Saturnino de Oliveira, Wêndeo Kennedy Costa, Adrielle Zagmignan, Luís Cláudio Nascimento da Silva, Magda Rhayanny Assunção Ferreira, Vilmar Luiz Lermen, Maria Silvanete Benedito de Sousa Lermen, Alexandre Gomes da Silva, Rafael Matos Ximenes, Luiz Alberto Lira Soares, Patrícia Maria Guedes Paiva, Vera Lúcia de Menezes Lima, Maria Tereza dos Santos Correia, Márcia Vanusa da Silva

**Affiliations:** ^1^Departamento de Bioquímica, Universidade Federal de Pernambuco, Recife, PE 50670-901, Brazil; ^2^Programa de Pós-Graduação, Universidade Ceuma, São Luís, MA 65075-120, Brazil; ^3^Departamento de Ciências Farmacêuticas, Universidade Federal de Pernambuco, Recife, PE 50670-901, Brazil; ^4^Comunidade Serra dos Paus Dóias Chapada do Araripe, Exu, PE 56230-000, Brazil; ^5^Departamento de Antibióticos, Universidade Federal de Pernambuco, Recife, PE 50670-901, Brazil

## Abstract

This work evaluated the antioxidant properties and *in vivo* antinociceptive and anti-inflammatory effects of extracts obtained from fruit peels of *Myrciaria floribunda* (H. West ex Willd.) O. Berg (Myrtaceae). This plant is popularly known in Brazil as *Cambuí* or *camboim*. Different extracts were submitted to comparative analysis to determine the content of selected phytochemical classes (levels of total phenols, flavonoids, and monomeric anthocyanins) and the *in vitro* antioxidant potentials. The extract with higher potential was selected for *in vivo* evaluation of its antinociceptive and anti-inflammatory action. Finally, the chemical characterization of this extract was performed by high-performance liquid chromatography (HPLC). MfAE (extract obtained using acetone as solvent) showed the higher levels of phenols (296 mg GAE/g) and anthocyanins contents (35.65 mg Cy-3-glcE/g) that were associated with higher antioxidant activity. MfAE also exhibited *in vivo* anti-inflammatory and analgesic propertiers. This fraction inhibited the inflammatory and neurogenic phases of pain, and this effect was reversed by naloxone (suggesting the involvement of opioidergic system). MfAE reduced the abdominal contortions induced by acetic acid. The HPLC analysis revealed the presence of gallic acid (and its derivatives) and ellagic acid. Taken together, these data support the use of *M. floribunda* fruit peels for development of functional foods and nutraceutics.

## 1. Introduction

Free radicals (and associated reactive species) are in general derived from normal metabolism and are crucial for redox signaling pathways and immune defense [1–4]. However, these molecules can interact with cellular structures leading to impairment of physiological systems [5–7]. In this sense, the oxidative stress has been associated with the etiology of several pathologies such as inflammatory disorders, chronic pain, and degenerative diseases [8–10].

Inflammation is a complex condition triggered by several stimuli (mechanical injuries, toxic compounds, tissue ischemia, and infectious agents) and marked by tissue alteration that allows the intense migration of immune cells to the inflammatory focus [11–13]. Usually, the release of proinflammatory substances (cytokines, nitric oxide, and other reactive species) is reduced after the eradication of stimuli, leading to the resolution phase [13, 14]. However, in some situations, the resolution of inflammation is not achieved, resulting in excessive or inappropriate inflammation [11]. This condition is associated with several diseases such as cancer, diabetes, rheumatoid arthritis, and psoriasis [12, 15]. In this case, the high levels of reactive species lead to tissue damage and organ dysfunction [2, 4, 5].

The excessive release of inflammatory mediators can result in the establishment of inflammatory pain [16, 17]. For example, the cytokines TNF-*α*, IL-1*β*, and IL-6 (produced by macrophages and other immune cells) interact with nociceptive neurons and modulated central sensitization, pain, and hyperalgesia [18–20]. Furthermore, other substances produced by immune cells, such as histamines and prostaglandins, also play important roles in pain regulation [17, 21, 22]. In this context, pain and inflammation have a tightly regulated relationship, since the nociceptive neurons also regulate the inflammatory response [17, 21, 23].

Despite the number of anti-inflammatory and analgesic drugs available in the market, the treatment schedules of these clinical conditions are complex and pose huge challenges to the health systems worldwide [24, 25]. This scenario points out the urgent need for the development of effective analgesic and anti-inflammatory drugs [26–28].

Several scientific evidences report that fruits are valuable sources of compounds with antioxidant properties, and their consumption may be beneficial to reduce the deleterious effects of inflammatory disorders and chronic pain [29–32]. In this sense, there is a growing interest in knowing the anti-inflammatory and analgesic properties of edible fruits (and their different parts) in order to provide more insights into supporting their use as functional foods and nutraceuticals [30, 33, 34].

*Myrciaria floribunda* (H. West ex Willd.) O. Berg (Myrtaceae) is native plant of South and Central America, which is distributed in different Brazilian biomes such as Amazon, Caatinga, Cerrado, and Atlantic Forest. Its fruits (known in Brazil as *Cambuí* or *camboim*) are edible and often consumed freshly, in juices or alcoholic beverages [35]. Some works have shown the pharmacological properties of different parts of this plant such as antioxidant, antimicrobial, and anticancer effects [36, 37]. However, the pharmacological actions of the fruits were not properly addressed [35]. In particular, the beneficial aspects of the products derived from fruit peels of the *M. floribunda* have not been demonstrated. This work analyzed the phytochemical composition along with the antioxidant, antinociceptive, and anti-inflammatory potentials of extracts obtained from fruit peels of *M. floribunda*.

## 2. Materials and Methods

### 2.1. Plant Material

Fruits of *M. floribunda* were collected from *Serra dos Paus Dóias* (Exu, Pernambuco, Brazil) in 2015. The plant material was identified by Alexandre Gomes da Silva (Department of Antibiotics, Federal University of Pernambuco, Brazil). The specimen voucher (number: 92722) was deposited in the Herbarium of the Agronomic Institute of Pernambuco.

### 2.2. Plant Extracts

The fruits were washed with running water and manually separated into pulp, seed, and peel. Samples of the fruit peels were dried at 40°C and reduced to fine powder. The fruit peel powder (50 g) was extracted with 500 mL of ethyl ether (1 : 10, w/v) in brown glass bottles with cap by vigorous shaking for 1 hour at room temperature (30°C). The extract was then filtered, and the residue was re-extracted twice more following the same procedure with 500 mL. After this process, the residue was extracted with other solvents (in the sequence chloroform, acetone, and methanol), and all extraction steps were repeated as described above. At each step, the obtained extracts were dried in a rotaevaporator at 40°C, generating extracts MfEeE (ethyl ether), MfCE (chloroform), MfAE (acetone), and MfME (methanol). Aqueous extraction was also performed with 10 g of the powder with 100 mL of distilled water (1 : 10, w/v); then, the solution was filtered and lyophilized, generating the extract MfAqE.

### 2.3. Phytochemical Analysis

The presence of several kinds of phytochemicals (flavonoids, phenylpropanoids, triterpenes, steroids, saponins, monoterpenes, sesquiterpenes, coumarins, quinones, alkaloids, proanthocyanidins, and water-soluble tannins) was investigated in each extract using thin-layer chromatography with silica gel plate F_254_ (ALUGRAM^R^ 818131, Macherey-Nagel, Germany), and for each elution system, specific visualization agents and standards were used to identify the metabolites as described in Table 1 [38–41].

### 2.4. Total Phenol Content, Total Flavonoid, and Monomeric Anthocyanins

The levels of total phenols were estimated using the Folin–Ciocalteu method [42], with some modifications. Samples of 20 *μ*L of each extract (1 mg/mL) were mixed with 100 *μ*L of the Folin–Ciocalteu reagent. After 3 minutes at room temperature, 80 *μ*L of a sodium bicarbonate solution (0.7 M) was added. The reaction was kept in the dark for 2 hour at room temperature. The absorbance was measured at 735 nm using a microplate reader (BioTek uQuant MQX200). Methanol and distilled water were used as negative controls. Gallic acid was used as standard, and the results were calculated based on the calibration curve of gallic acid (10–100 *μ*g/mL) and expressed as mg equivalent of gallic acid per gram of extract (GAE/g extract).

The flavonoid content was determined according to the colorimetric method of aluminum chloride [43]. The extracts were tested at the concentration of 1 mg/mL, and quercetin was used to obtain the standard calibration curve (10–100 *μ*g/mL). The sample (100 *μ*L) was mixed with 100 *μ*L of the reagent [2% aluminum chloride (AlCl_3_) in methanol]; after 1 hour in dark-room-temperature environment, absorbance was read against a blank of methanol or more distilled water, the reagent, at 420 nm. The results obtained were expressed as mg equivalent of quercetin per gram of extract (mg QE/g extract). The experiment was done in triplicate.

The content of monomeric anthocyanins was determined by the differential pH method [44]. Potassium chloride buffer (KCl; 0.025 M; pH 1) and sodium acetate buffer (CH_3_CO_2_Na; 0.4 M; pH 4.5) were used. The sample (200 *μ*L at 10 mg/mL) was mixed to 1.8 mL of each buffer. After 10 minutes, the absorbance was read at 520 nm and at 700 nm. The distilled water was used as control. The concentration of anthocyanin was calculated according to the following equation, and the results were expressed as cyanidin-3-glucoside equivalent (Cy-3-glcE):

(1) $$\begin{array}{c} \text{anthocyanin content}=\frac{\left(A\times \text{MW}\times \text{DF}\times \;10^{3}\right)}{\varepsilon \times 1}. \end{array}$$

*A* = (absorbance 520 nm−absorbance 700 nm) at pH 1 −(absorbance 520 nm−absorbance 700 nm) at pH 4.5; MW (molecular weight) = 449.2 g/mol of Cy-3-glc; DF = dilution factor (0.2 mL of the sample was diluted to 2 mL, DF = 10); 1 = length in cm; *ε* = 26900, molar extinction coefficient, in L x mol^−1^ x cm^−1^, for Cy-3-glc; and 10³ = factor for conversion from g to mg.

### 2.5. Antioxidant Assays

#### 2.5.1. DPPH Assay (Free Radical Sequestration)

For evaluation of the ability to sequester the DPPH (2,2-diphenyl-1-picryl-hydrazyl) radical, an aliquot of 40 *μ*L of each extract at different concentrations (8.0625 to 1000 *μ*g/mL) was mixed with 250 *μ*L of 1 mM DPPH solution (in methanol) for 25 minutes at room temperature and protected from light [45]. Gallic acid was the standard used as a positive control, and methanol was the negative control. A control solution (40 *μ*L of the extract and 250 *μ*L of the solvent used to dilute the samples) was used to rule out the possible interference of extract color. The absorbance of each solution was measured at 517 nm using a microplate reader (BioTek uQuant MQX200).

#### 2.5.2. ABTS Assay

The ABTS (2,2-azino-bis (3-ethylbenzothiazoline-6-sulfonic acid)) radical was obtained by mixing 5 mL of the ABTS stock solution (7 mM) with 88 *µ*L of 140 mM potassium persulfate solution. This mixture remained in the dark and at room temperature for 16 hours before use. The ABTS radical solution was diluted with ethanol until an absorbance of 0.7 nm (±0.02) was obtained at 734 nm. Samples (10 *μ*L) of the extracts (1 mg/mL) were mixed with 1 mL of the ABTS radical for 6 minutes and then read off a spectrophotometer at 734 nm [46]. Trolox was used as standard antioxidant (100 *μ*M to 2000 *μ*M). The result was expressed as Trolox equivalent antioxidant capacity (TEAC) in *μ*M TE/g extract.

#### 2.5.3. Total Antioxidant Capacity (TAC)

Samples (100 *μ*L) of the extracts (1 mg/mL) were combined with 1 mL of the reagent solution (0.6 M sulfuric acid, 28 mM sodium phosphate, and 4 mM ammonium molybdate). The tubes were incubated at 95°C for 90 minutes and then cooled to room temperature, and the absorbance of each reaction was read at 695 nm. The control reaction consisted in 100 *μ*L of methanol mixed with 1 mL of the reagent solution [47]. The standard used was ascorbic acid (1 mg/mL). The result was calculated according to the formula below and was expressed as total antioxidant capacity (TAC) relative to ascorbic acid.

(2) $$\begin{array}{c} \text{TAC}\%=\frac{S_{\text{abs}}-B_{\text{abs}}}{{\text{AA}}_{\text{abs}}-B_{\text{abs}}}*100, \end{array}$$

where *S*_abs_ is the absorbance of the sample (extracts), *B*_abs_ is the absorbance of control, and AA_abs_ is the absorbance of ascorbic acid.

#### 2.5.4. Determination of the Sequestration of the Superoxide Radical

In this assay, the reaction mixture consisted of 300 *μ*L of extracts at different concentrations (50–1000 *μ*g/mL), 100 *μ*L of NBT (nitrotetrazolium blue chloride) (1 mg/mL in DMSO solution), and 1 mL of alkaline DMSO (1 mL DMSO containing 5 mM sodium hydroxide in 0.1 mL water), and the absorbance was measured at 560 nm [48].

### 2.6. HPLC Analysis

For high-performance liquid chromatography (HPLC) analysis, 20 mg of the MfAE was weighed and diluted with methanol 50% (v/v) to a volumetric flask of 2 mL and filtered through a 0.45 *μ*m PVDF membrane (Macherey-Nagel®) for sample injection. The system was the Ultimate 3000 (Thermo Fisher Scientific®, EUA) coupled to a photodiode array detector (DAD; Thermo Fisher Scientific®) and equipped with a binary pump (HPG-3x00RS; Thermo Fisher Scientific®), degasser, and automatic sampler loop volume of 20 *μ*L (ACC-3000; Thermo Fisher Scientific). The wavelength for the analyses was set at 270 nm.

The chromatographic separation was performed at 26°C using a column Dionex® C_18_ (250 mm × 4.6 mm d.i., 5 *μ*m) equipped with precolumn Phenomenex® (C_18_; 4 mm × 3.9 *μ*m). The mobile phase consisted of solvent A (purified water, Purelab Classic UV, Elga®) and solvent B (methanol, HPLC grade, Tedia®), both acidified with 0.05% trifluoracetic acid (Vetec®), and the flow rate was adjusted to 0.9 mL/minute. The following gradient program was used: 0–10 minutes, 10–20% of solvent B; 10–13.5 minutes, 20–25% of solvent B; 13.5–18 minutes, 25–40% of solvent B; 18–25 minutes, 40–80% of solvent B; 25–30 minutes, 80% of solvent B; and 30–35 minutes, 80–10% of solvent B. The content of the substances found in MfAE was determined from the calibration curves using gallic acid (96% of purity) or ellagic acid (95% of purity), both purchased from Sigma^®^ (USA).

### 2.7. Ethical Statement

All procedures using animals were approved by the Ethics Committee on the Use of Animals of the Federal University of Pernambuco, Brazil (CEUA-UFPE; Process Number: 0003/2018). The proposal was approved in the meeting of CEUA-UFPE held in April 23, 2018.

### 2.8. Animals

The experiments were conducted using male Swiss mice (30–35 g, 10 weeks) supplied by the Laboratory of Immunopathology Keizo Asami (LIKA). The animals received food and water standard ad libitum with light/dark period of 12 h. Before each experiment (6–8 hours), animals were limited to a water-only diet to avoid foodborne interference with substance absorption.

### 2.9. Evaluation of Acute Toxicity

Acute toxicity of MfAE was performed according to the instructions of the Organization for Economic Cooperation and Development (2001). The acute toxicity of MfAE was analyzed in two steps. Phase 1: animals were divided into three groups (*n* *=* *3*) that received MfAE at doses of 10 mg/kg, 100 mg/kg, and 1000 mg/kg. The animals are placed under observation for 24 hours to monitor their behavior and survival. Phase 2: MfAE was administered at higher doses (1600, 2900, and 5000 mg/kg; one mice per dose), and then the behavior and survival were observed for 24 hours [49]. The LD50 was calculated by the following equation:

(3) $$\begin{array}{c} {\text{LD}}_{50}=\sqrt{\left(D_{0}\times D_{100}\right)}, \end{array}$$

*D*_0_ = highest dose that did not induce mortality and *D*_100_ = lowest dose that resulted in mice mortality.

### 2.10. Anti-Inflammatory Assay

The *in vivo* anti-inflammatory action of MfAE was evaluated by carrageenan-induced paw edema. For this, mice were allocated into four groups (*n* *=* *6*) that received the following treatments: (i) MfAE at 50 mg/kg (*p.o.*), (ii) MfAE at 100 mg/kg (*p.o.*), (iii) vehicle (0.9% saline solution) (*p.o.*), or (iv) indomethacin at 20 mg/kg (*p.o.*). After 1 h, paw edema was induced by an injection of 2% carrageenan solution (15 *μ*L/animal) into the subplantar region of the right hind paw [50]. As control, saline solution (0.9%; 15 *μ*L) was injected into the left hind paw. Paw volume was measured using digital Vernier caliper at each hour for 5 hours [51]. Inhibition of edema was calculated by evaluating the difference between the volumes of right and left paws.

### 2.11. Antinociceptive Assays

#### 2.11.1. Abdominal Pain Induced by Acetic Acid

The mice were divided into four groups (*n* *=* *6*) and were treated as described above. The nociception was induced by an intraperitoneal injection of acetic acid (0.8%; 100 mL/10 g). Starting 5 minutes after acetic acid injection, the number of abdominal contortions was recorded for 10 minutes by the number of stretching movements [52].

#### 2.11.2. Formalin Assay

In this assay, the animals were divided into five groups (*n* *=* *6*/group) that received the following treatments: (i) vehicle, (ii) MfAE at 50 mg/kg (*p.o.*), (iii) MfAE at 100 mg/kg (*p.o.*), (iv) indomethacin at 20 mg/kg (*i.p.*), and (v) morphine at 10 mg/kg (*p.o.*). After 1 h, the formalin solution (2.5% in 0.9% saline; 20 *μ*L/paw) was administered into subplantar area of the right hind paw [53]. Mice were observed in a chamber with a mirror mounted onto three sides to allow the observation of the paws. The time (in seconds) spent for licking and biting the injected paw was measured as an indicator of pain. This response was measured for 5 minutes (first phase, neurogenic pain) and between 15 and 30 minutes after formalin injection (second phase, inflammatory pain).

To evaluate the possible involvement of the opioid system in the antinociceptive effect of MfAE, the mice were treated with naloxone (1 mg/kg, *i.p.*) 30 minutes prior to administration of MfAE (100 mg/kg, *p.o.*) and morphine (10 mg/kg, *p.o.*).

### 2.12. Statistical Analysis

The *in vitro* assays were performed in triplicate in at least two independent assays. The results were expressed as mean (±standard error). The difference between means was tested for statistical significance using one-way or two-way analysis of variance (ANOVA) followed by Tukey's test for multiple comparisons. The IC_50_ (concentration that inhibits 50%) was calculated by linear regression. In the statistical analysis of the results, the value of *p* < 0.05 was considered statistically significant.

## 3. Results and Discussion

### 3.1. Phytochemical Analysis

The yield of each extract was 8.50%, 6.60%, 2.05%, 5.17%, and 49.27% for MfAqE, MfEeE, MfCE, MfAE, and MfME, respectively. The chromatographic profile of the extracts from *M. floribunda* fruit peels revealed the presence of several classes of secondary metabolites (Table 2). In the MfEeE and MfCE, monoterpenes, triterpenes, sesquiterpenes, and steroids were found. MfAE is composed by flavonoids, phenylpropanoids, traces of triterpenes and steroids, saponins, and hydrolysable tannins. On the other hand, MfME and MfAqE have flavonoids, polymeric proanthocyanidins, and traces of hydrolysable tannins.

Some classes of secondary metabolites detected in the extracts from fruit peels of *M. floribunda* have been already described for the genus *Myrciaria*, such as steroids, proanthocyanidins, flavonoids, and hydrolysable tannins [36, 37, 54]. In addition, the edible parts of the *M. floribunda* fruits are composed by carotenoids (lutein, zeaxanthin, *β*-cryptoxanthin, 13-*cis*-*β*-carotene, *α*-carotene, *β*-carotene, and 9-*cis*-*β*-carotene), flavonoids (rutin), and phenolic acids (gallic acid and ellagic acid) [35].

### 3.2. Comparative Analysis of the Concentration of Total Phenols, Flavonoids, and Monomeric Anthocyanins

In order to select the extract with the highest potential, we performed a comparative evaluation of the amount of phenols, flavonoids, and monomeric anthocyanins. The content of total phenols varied among the extracts from 7.54 mg GAE/g to 296.27 mg GAE/g (Table 3). The highest phenol content was obtained for MfAE (296.27 mg GAE/g) (Table 3).

The levels of total flavonoid content ranged from 0.74 mg QE/g to 16.62 mg QE/g (Table 3). The MfEeE showed the highest concentration (16.62 mg QE/g). Finally, the content of monomeric anthocyanins ranged from 4.34 mg Cy-3-glcE/g to 35.65 mg Cy-3-glcE/g (Table 3) and MfAE showed the highest content (35.65 Cy-3-glcE/g). In summary, MfAE showed the highest content of phenolic compounds and anthocyanins. These classes of compounds have been associated with several beneficial properties of foods and medicinal plants [55–57].

### 3.3. Comparative Evaluation of Antioxidant Activity

Following, four antioxidant assays were employed to select the extract with the best potential (Table 4). In the DPPH assay, the lower IC_50_ value was found for MfAE (63.84 *μ*g/mL), followed by MfME (343.12 *μ*g/mL), MfAqE (350.41 v), and MfEeE (854.94 *μ*g/mL). Similarly, MfAE showed the highest ability to inhibit the ABTS radical (1630.11 *µ*M TEAC/g) and total antioxidant capacity (123.91%). Finally, only MfAE and MfME were able to sequester the superoxide radical, with IC_50_ values of 260.27 *μ*g/mL and 446.31 *μ*g/mL, respectively (Table 4).

The highest antioxidant action exhibited by MfAE may be explained by its higher content of anthocyanins and phenolic compounds, since the levels of these phytocompounds showed positive correlations with the results obtained in the *in vitro* antioxidants assays (Table 4). In addition, MfAE presented a phytochemical profile with different classes of secondary metabolites, such as flavonoids, phenylpropanoids, triterpenes, saponins, and hydrolysable tannins. Taken together, the results suggest that MfAE is the most promising extract, leading us to select it for the *in vivo* anti-inflammatory and antinociceptive evaluation.

### 3.4. HPLC Analysis of the Acetone Extract of *M. floribunda*

**Figure 1:**
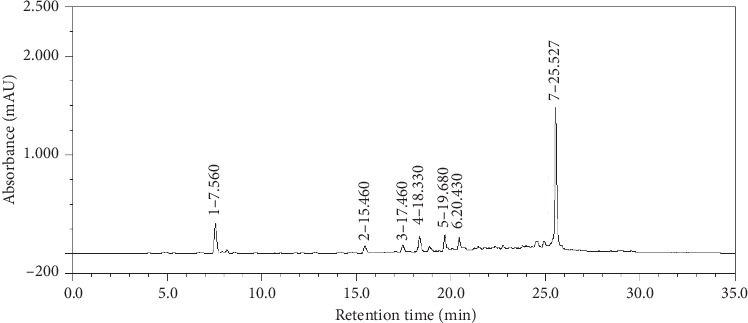
Chromatographic profile of the phenolic acids from acetone extract (MfAE). The extract was analyzed by HLPC at 270 nm. Detected compounds: (1) gallic acid, (2)–(6) gallic acid derivatives, and (7) ellagic acid.

The chromatographic profile of the acetone extract from peels of the fruit (MfAE) is shown in Figure 1. The analysis revealed the presence of gallic acid (peak 1, with retention time of 7.56 minutes) and an indicative presence of ellagic acid (peak 7, with retention time 25.52 minutes). The other peaks (2 to 6) are gallic acid derivatives. The contents of the compounds were calculated as follows: 0.29 ± 0.0012 g% gallic acid, 0.33 ± 0.0204 g% gallic acid (derivatives, peaks of 2 to 6), and 0.54 ± 0.0012 g% ellagic acid. The evidenced peaks(gallic acid and ellagic acid) were identified by comparing the retention times and UV spectra of standards. Also, they were confirmed by spiking the sample with a small amount of the standards solutions. Both gallic acid and ellagic acid were previously detected in the fruit of *M. floribunda* [35].

Gallic acid and ellagic acid (and their derivatives) are usually found in fruits and other plant-derived products [55, 58] with antioxidant activity, including those from *Myrciaria* genus [59, 60]. Furthermore, some works have reported that these compounds are potent anti-inflammatory, analgesic, and neuroprotective agents [60–62].

### 3.5. Acute Toxicity

The oral lethal dose (LD_50_) of acetone extract was greater than 2000 mg/kg (LD_50_ = 2154.06 mg/kg), therefore, it can be classified as a low toxic agent according to the Organization for Economic Cooperation and Development [63]. Thus, the doses of 50 mg/kg and 100 mg/kg were selected to be applied in the *in vivo* assays.

### 3.6. Anti-Inflammatory Activity

**Figure 2:**
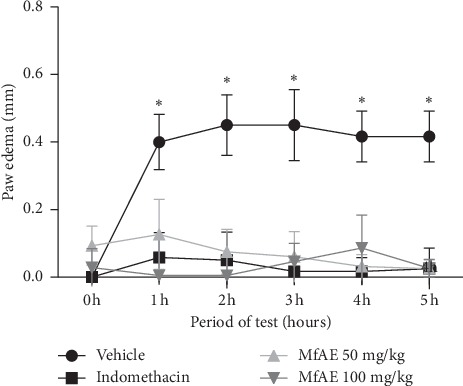
Effect of the acetone extract from fruit peels of *Myrciaria floribunda* (MfAE, 50 and 100 mg/kg, v.o.) on carrageenan-induced paw edema. Each point represents the mean ± SEM of the six animals. Asterisks indicate significance compared with vehicle group. ^*∗*^*p* < 0.001, two-way ANOVA.

The *in vivo* anti-inflammatory of MfAE was evaluated in a model where the formation of edema was induced by carrageenan. The animals treated with both doses of MfAE demonstrated significant reductions of paw edema, in all periods evaluated, when compared to vehicle-treated groups (*p* < 0.001) (Figure 2). Maximum inhibition (98.87%) was observed for 2 hours of treatment with MfAE at 100 mg/kg. For the dose of 50 mg/kg, the higher effect (93.75%) was observed after 5 hours. The reference drug indomethacin (20 mg/kg) exhibited maximum inhibition (96.22%) after 3 hours of treatment.

Carrageenan-induced paw edema is an experimental model useful for the evaluation of new agents acting on acute inflammation [64–66]. This test is considered a biphasic event; in the initial phase (1 to 3 hours), the release of inflammatory mediators such as histamine, serotonin, and bradykinin occurs, while in the late phase (3 hours to 4 hours), the synthesis of cyclooxygenase 2 (COX-2) isoform occurs, leading to prostaglandins and nitric oxide release [67, 68]. MfAE reduced the edema during the five hours observed, suggesting that it acts in both phases of the acute inflammatory process. Further, extracts from other *Myrciaria* plants also exhibited anti-inflammatory action in this model [64, 65].

The results suggest that the effect of MfAE in the formation of edema may involve multiple points such as the inhibition of the synthesis and/or release of inflammatory mediators, via COX or different specific enzymatic mechanism. The richness of polyphenols (flavonoids, anthocyanins) in MfAE has contributed towards the anti-inflammatory activity [60]. However, complementary studies based on these inflammatory mediators and COX inhibition should be explored, as well as phytochemical isolation.

### 3.7. Antinociceptive Activity

**Figure 3:**
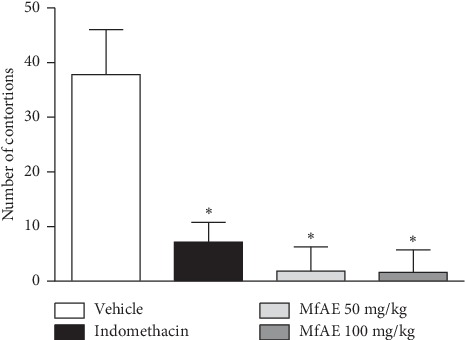
Effect of the acetone extract of the fruit peels of *Myrciaria floribunda* (MfAE, 50 and 100 mg/kg, v.o.) on the abdominal contortion induced by acetic acid. Each column represents the mean ± SEM of the number of contortions of the six animals. Asterisks indicate significance compared with vehicle group. ^*∗*^*p* < 0.001, one-way ANOVA.

The treatment with both doses of MfAE also significantly inhibited the number of contortions (95.16% and 95.59%, resp.), compared to vehicle (saline) (*p* < 0.001). There were no differences neither between the different concentrations, nor in relation to the indomethacin control (Figure 3). In this test, acetic acid induces nociception through a mechanism that depends on the resident cells (macrophages and mast cells) presented at the peritoneal cavity. These cells release cytokines (TNF-*α*, interleukin 1*β*, and interleukin 8) that are involved in the contortion response [69]. However, this model cannot infer whether the antinociceptive action of MfAE is central or peripheral.

**Figure 4:**
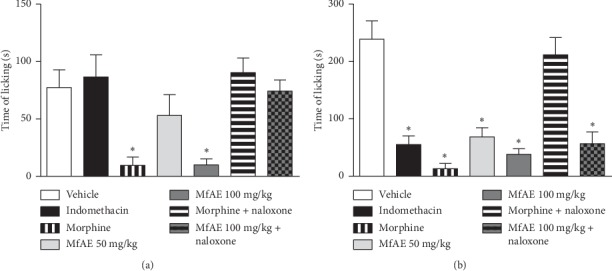
Effect of the acetone extract of the fruit peels of *Myrciaria floribunda* (MfAE, 50 and 100 mg/kg, v.o.) on the formalin test. Stage 1: 0 to 5 minutes of test, neurogenic phase; Stage 2: 15 to 30 minutes of test, inflammatory phase. Each column represents the mean ± SEM of the six animals. Asterisks indicate significance compared to the vehicle group. ^*∗*^*p* < 0.001, one-way ANOVA.

Regarding the formalin test, the results shown in Figure 4 reveal that in the first phase (neurogenic phase) of the test, only the concentration of 100 mg/kg showed a significant reduction of licking (86.52%; *p* < 0.001). Morphine (10 mg/kg) induced a reduction of 83.98% at this stage. However, in the second phase (inflammatory phase), both concentrations of MfAE had a significant reduction in licking time (*p* < 0.001). The inhibition was 69.47% and 82.58% for MfAE at 50 mg/kg and 100 mg/kg, respectively. Positive controls, indomethacin (20 mg/kg) and morphine (10 mg/kg), inhibited 76.45% and 94.91% of licking, respectively. Naloxone reverted the effects of MfAE only in the neurogenic phase.

The formalin test is a nociception model based on a biphasic pain response; it is a useful tool to investigate whether the action of new agents has an analgesic and anti-inflammatory effect [70]. In the first phase (neurogenic pain) which occurs up to 5 minutes after the injection of formalin in the paw, the direct chemical stimulation on nociceptors activates the afferent fibers and leads to the release of substance P. In the inflammatory phase (15 to 30 minutes after the injection), the release of inflammatory mediators occurs, triggering a peripheral inflammatory response [53, 71].

Centrally acting analgesic drugs (such as morphine) are able to inhibit both phases of the formalin test; in contrast, peripherally acting drugs (such as indomethacin) inhibit only the second phase [72]. Our results demonstrate that MfAE at the dose of 100 mg/kg can reduce both phases of the nociceptive response (*p* < 0.001). In the first phase, the analgesic effect of MfAE (100 mg/kg) was mediated by the action on the opioid receptors, as naloxone reversed the effect of MfAE. This observation is attributed to the opioidergic mechanism that has been antagonized in the administration of naloxone (selective opioid receptor antagonist).

## 4. Conclusion

The results of this work show that the extracts from the peels of *M. floribunda* fruits are sources of compounds with antioxidant activity. The use of fruit peels, a possible residue of fruit consumption, represents a sustainable application of these natural products. In addition, the comparative analysis of antioxidant and phytochemical profiles allowed the selection of MfAE (a fraction rich in phenolic compounds and anthocyanins) as an agent high in pharmacological potential. MfAE also showed significant anti-inflammatory and antinociceptive activities (effect involving the opioidergic system). Considering the present results, the fruit peels of *M. floribunda* are sources of high-value bioactive compounds that may contribute to several beneficial effects associated with the consumption of this fruit. In this sense, the fruit peels can be used for development of functional foods and nutraceuticals.

## Figures and Tables

**Table 1:** Elution systems, chromogenic agents, and standards used in the phytochemical analysis of extracts from fruit peels of *Myrciaria floribunda* with thin-layer chromatography (TLC).

Secondary metabolite classes	Standard	Mobile phase	Chromogenic agent
Flavonoids and phenylpropanoids	Quercetin, rutin, and chlorogenic acid	EtOAc-HCOOH-AcOH-H_2_O (100 : 11 : 11 : 26 v/v)	Natural products-polyethylene glycol reagent

Triterpenes and steroids	*β*-Sitosterol	Toluene:EtOAc (90 : 10 v/v)	Liebermann–Burchard reagent

Saponins	Escin	EtOAc-HCOOH-AcOH-H_2_O (100 : 11 : 11 : 26 v/v)	Liebermann–Burchard reagent

Mono and sesquiterpenes	Thymol	Toluene:EtOAc (97 : 3 v/v)	Anisaldehyde-sulfuric acid reagent

Coumarins and quinones	Coumarin and lapachol	CHCl_3_-MeOH (98 : 2 v/v)	Potassium hydroxide reagent

Alkaloids	Pilocarpine	EtOAc-HCOOH-AcOH-H_2_O (100 : 11 : 11 : 26 v/v)	Dragendorff reagent

Proanthocyanidins	Catechin	EtOAc-HCOOH-AcOH-H_2_O (100 : 11 : 11 : 26 v/v)	Vanillin-hydrochloric acid reagent

Hydrolysable tannins	Gallic acid	*n*-BuOH-H_2_O-AcOH (40 : 50 : 10 v/v)	Ferric ammonium sulfate 1%

**Table 2:** Phytochemical analysis of extracts from fruit peels of *Myrciaria floribunda.*

Class of secondary metabolites	Extracts from fruit peels of *Myrciaria floribunda*				
	MfEeE	MfCE	MfAE	MfME	MfAqE
Flavonoid	−	−	+^*∗*^	+^*∗*^	+^*∗*^
Phenylpropanoid	−	−	+	−	−
Triterpene	+	+	tr	−	−
Steroid	+	+	tr	−	−
Saponin	−	−	+	−	−
Monoterpene and sesquiterpene	+	+	−	−	−
Alkaloid	−	−	−	−	−
Coumarin	−	−	−	−	−
Quinone	−	−	−	−	−
Proanthocyanidin and leucoanthocyanidin	−	−	−	+^*∗∗*^	+^*∗∗*^
Hydrolysable tannin	−	−	+	+	+

−: negative; +: positive. *Note. *^*∗*^3′,4′-OH flavonoids (aglycones, mono-, di-, and triglycosides). ^*∗∗*^Polymeric proanthocyanidins.

**Table 3:** Concentration of total phenols, total flavonoids, and monomeric anthocyanins in different extracts obtained from fruit peels of *M. floribunda*.

Samples	Total phenol content (mg GAE/g)	Total flavonoid content (mg QE/g)	Anthocyanin content (mg Cy-3-glcE/g)^3^
MfEeE	7.54	16.62	ND
MfCE	ND	3.46	ND
MfAE	296.27	0.74	35.65
MfME	158.29	ND	4.34
MfAqE	62.28	ND	14.42

ND = not detected.

**Table 4:** Antioxidant activity of the extracts obtained from fruit peels of *Myrciaria floribunda*.

Samples	DPPH IC_50_ (*µ*g/mL)	ABTS^+^ (*µ*M TEAC/g)	TAC (%)	Superoxide IC_50_ (*µ*g/mL)
MfEeE	854.94	597.89	109.45	>1000
MfCE	>1000	347.89	68.87	>1000
MfAE	63.84	1630.11	107.43	260.27
MfME	343.12	660.11	102.82	446.31
MfAqE	350.41	793.44	123.91	NT
Gallic acid	3.46	—	—	—

IC_50_ = concentration required to reduce 50% of the DPPH or superoxide radical. TEAC = Trolox equivalent antioxidant capacity. NT = not tested.

## Data Availability

The data that support the findings of this study are available from the corresponding author upon reasonable request.
